# Prone positioning under VV-ECMO in SARS-CoV-2-induced acute respiratory distress syndrome

**DOI:** 10.1186/s13054-020-03162-4

**Published:** 2020-07-14

**Authors:** Bruno Garcia, Nicolas Cousin, Claire Bourel, Mercé Jourdain, Julien Poissy, Thibault Duburcq, on behalf of the Lille Intensive Care COVID-19 group, Pauline Boddaert, Arthur Durand, Ahmed El Kalioubie, Patrick Girardie, Marion Houard, Geoffrey Ledoux, Anne Sophie Moreau, Christopher Niles, Saad Nseir, Thierry Onimus, Aurelia Toussaint, Sebastien Préau, Laurent Robriquet, Anahita Rouze, Arthur Simonnet, Sophie Six, Morgan Caplan, Julien Goutay, Emmanuelle Jaillette, Erika Parmentier-Decrucq, Raphael Favory, Daniel Mathieu, Guillaume Degouy, Mouhamed Moussa

**Affiliations:** 1 CHU Lille, Pôle de réanimation, F-59000 Lille, France; 2 Univ. Lille, Inserm Pasteur Lille, Inserm U1190 EGID, F-59000 Lille, France; 3 Univ. Lille, Inserm U1285, CNRS, UMR 8576 - Unité de Glycobiologie Structurale et Fonctionnelle, F-59000 Lille, France

**Keywords:** COVID-19, Prone positioning, ECMO, Refractory hypoxemia

## Background

Infection due to severe acute respiratory coronavirus 2 (SARS-CoV-2) may lead to an atypical acute respiratory distress syndrome (ARDS) [1], requiring in the most severe cases veno-venous extracorporeal membrane oxygenation (VV-ECMO). The management of persistent severe hypoxemia under VV-ECMO requires a multi-step clinical approach including prone positioning (PP), which could improve oxygenation [2].

## Methods

We performed a retrospective study of patients with SARS-CoV-2-induced ARDS submitted to PP during VV-ECMO. We aimed to describe mechanical ventilation parameters and gas exchanges before and after PP. We assess the safety of PP and compare patients with PP under ECMO (prone ECMO group) to those maintained in the supine position (supine ECMO group). Patients were treated in accordance with the recommendation guidelines on ARDS [3]. During VV-ECMO, PP was considered in case of severe hypoxemia (PaO_2_/FiO_2_ ratio below 80 mmHg) despite FDO_2_ and FiO_2_ both at 100% and in case of extensive lung consolidation (ECL) on chest imaging (> 50% of lung volume).

## Results

We enrolled 208 COVID-19 patients. Among the 125 patients with ARDS, 25 (20%) required VV-ECMO, and 14 (56%) were placed at least once in PP for a total of 24 procedures with a median duration of 16 (15–17) h. The delay from ECMO implantation therapy to PP was 1.5 days [1–3]. The resultant changes in ventilator/ECMO settings and blood gas analysis before and after PP are displayed in Table 1. The median PaO_2_/FiO_2_ ratio improvement after PP was 28% [2–36]. High responders (increase PaO_2_/FiO_2_ ratio > 20%) were 62.5%, moderate-responders (increase PaO_2_/FiO_2_ < 20%) were 16.7%, and non-responders (decrease PaO_2_/FiO_2_) were 20.8%. We did not observe any major safety concerns but only pressure sores after 6 procedures, three minor hemorrhages at the injection cannula, and three moderate drops in VV-ECMO flow requiring fluid resuscitation. Pre-ECMO characteristics, ventilator/ECMO settings, and outcomes are exposed in Table 2. Patients in the prone ECMO group were less likely to be weaned from ECMO, and 28-day mortality rate was significantly higher.

**Table 1:** The resultant changes in ventilator/ECMO settings and blood gas analysis before and after PP

Variables	Before prone position	After prone position	***P*** value
**Mechanical ventilation settings**			
Tidal volume (mL/kg)	2.4 (1.8–2.9)	2.4 (1.8–2.7)	0.42
RR (breaths/min)	20 (16–25)	19 (16–25)	0.87
Plateau airway pressure (cmH_2_O)	28 (26–32)	29 (28–32)	0.43
PEEP (cmH_2_O)	14 (12–18)	16 (12–20)	0.36
Respiratory system compliance (mL/cmH_2_O)	18.6 (13.7–25.9)	17.9 (12.8–26.5)	0.92
Driving pressure (cmH_2_O)	14.5 (12–16.5)	14.5 (11–16)	0.56
Inspired fraction of oxygen (%)	70 (60–100)	67.5 (52.5–95)	0.16
**ECMO settings**			
ECMO blood flow (L/min)	6.2 (6–6.7)	6 (5.8–6.7)	0.41
Sweep gas flow (L/min)	7 (6–8.5)	7 (6.8–9)	0.15
FDO_2_ (%)	100 (80–100)	100 (80–100)	0.56
**Gas analysis**			
PaO_2_ (mmHg)	64 (51–78)	82 (66–109)	**0.007**
PaO_2_/FiO_2_ (mmHg)	84 (73–108)	112 (83–157)	**0.002**
PaCO_2_ (mmHg)	44 (41–46)	42 (36–49)	0.27
pH	7.38 (7.35–7.43)	7.38 (7.34–7.42)	0.47

Data are expressed as number (%) or median [IQR]. Analyses were performed with the GraphPad Prism 6 software (San Diego, CA). All tests were two-tailed, with *α* level at 0.05. The comparisons before vs. after prone position were realized by Wilcoxon matched-pairs signed-rank test

*RR* respiratory rate, *PEEP* positive end-expiratory pressure, *FiO*_*2*_ fraction of inspired oxygen, *PaCO*_*2*_ arterial partial pressure of carbon dioxide, *PaO*_*2*_ arterial partial pressure of oxygen, *FDO*_*2*_ fraction on oxygen delivered in the sweep gas, *ECMO* extracorporeal membrane oxygenation

**Table 2:** Pre-ECMO characteristics, ventilator/ECMO settings, and outcomes

	All patients (***n*** = 25)	Prone ECMO (***n*** = 14)	Supine ECMO (***n*** = 11)	***P*** value
Age (years)	59 (49.5–63)	59 (48–63)	57 (48–66)	0.82
Male sex, *n* (%)	22 (88)	12 (85.7)	10 (90.9)	1
Body mass index (kg/m^2^)	32 (28.4–37.5)	31.5 (28–38)	33.6 (28.4–37.6)	0.86
Comorbidities				
Any, *n* (%)	6 (24)	3 (21.4)	3 (27.3)	1
Hypertension, *n* (%)	12 (48)	6 (42.8)	6 (54.5)	0.7
Diabetes, *n* (%)	10 (40)	5 (35.7)	5 (45.4)	0.7
SAPS II	60 (40–65)	59.5 (46–62)	61 (38–80)	0.39
Delay symptoms-ECMO (days)	16 (11–18)	15 (11–20)	16 (10–16)	0.94
Delay mechanical ventilation-ECMO (days)	7 (4–10)	6.5 (4–10)	7 (4–13)	0.99
Chest imaging (X-rays or CT)				
Consolidation, *n* (%)	14 (56%)	11 (78.6)	3 (27.3)	**0.02**
Ground glass opacity, *n* (%)	25 (100)	14 (100)	11 (100)	–
Bilateral infiltration, *n* (%)	25 (100)	14 (100)	11 (100)	–
PaO_2_/FiO_2_ ratio before ECMO (mmHg)	84 (69–98)	84 (67–96)	87 (66–102)	0.77
Prone position before ECMO, *n* (%)	25 (100)	14 (100)	11 (100)	**–**
Neuromuscular blockers, *n* (%)	25 (100)	14 (100)	11 (100)	**–**
iNO before ECMO, *n* (%)	21 (84)	12 (85.7)	9 (81.8)	1
Corticosteroids, *n* (%)	4 (16)	1 (7.1)	3 (27.3)	0.29
**MV and ECMO settings the first day of ECMO**				
Tidal volume (mL/kg)	2.6 (1.9–2.9)	2.4 (1.7–3.1)	2.6 (2.1–2.8)	0.71
Plateau airway pressure (cmH_2_O)	26 (23–29)	26 (25–29)	26 (21–29)	0.52
PEEP (cmH_2_O)	14 (11–20)	14 (12–20)	14 (10–20)	0.47
Driving pressure (cmH_2_O)	10 (9–13)	11 (10–14)	9 (8–12)	0.44
Respiratory rate (cycles/min)	14 (12–18)	18 (13–25)	12 (12–14)	**0.006**
Respiratory system compliance (mL/cmH_2_O)	25 (15–32)	24 (14–30)	28 (18–33)	0.36
Inspired fraction of oxygen (%)	50 (50–80)	55 (50–72.5)	50 (40–80)	0.53
ECMO blood flow (L/min)	5.9 (5–6.3)	5.8 (5.2–6.7)	5.9 (4.9–6)	0.31
Sweep gas flow (L/min)	5 (4–6)	5.5 (4.5–6.2)	4 (3.5–5)	**0.04**
Membrane lung fraction of oxygen (%)	100 (80–100)	100 (80–100)	100 (90–100)	0.38
**Outcomes**				
ECMO weaning, *n* (%)	11 (44)	3 (21.4)	8 (72.7)	**0.02**
ECMO duration (days)	10 (5–13)	11 (6–13)	6 (3–12)	0.28
28-day mortality, *n* (%)	14 (56)	11 (78.6)	3 (27.3)	**0.02**
Discharged alive from ICU, *n* (%)	10 (40)	2 (14.3)	8 (72.7)	**0.005**
Still in ICU, *n* (%)	1 (4)	1 (7.1)	0	1

Data are expressed as number (%) or median [IQR]. Analyses were performed with the GraphPad Prism 6 software (San Diego, CA). All tests were two-tailed, with *α* level at 0.05. To compare the prone ECMO group to the supine ECMO group, we used the non-parametric Mann-Whitney test for continuous variables and the exact Fisher test for categorical ones

*SAPS II* Simplified Acute Physiology Score, *IQR* interquartile range, *CT* computed tomography, *PaO*_*2*_ arterial partial pressure of oxygen, *FiO*_*2*_ fraction of inspired oxygen, *PEEP* positive end-expiratory pressure, *ECMO* extracorporeal membrane oxygenation, *ICU* intensive care unit

## Discussion

We report that during VV-ECMO, PP improved oxygenation without a change in respiratory system compliance and PaCO_2_ at constant levels of minute ventilation and sweep gas flow. This does not suggest lung recruitment by PP but rather an optimization of ventilation and perfusion matching. Three explanations could be advanced for the mortality rate in the prone ECMO group (78.6%). First, prone ECMO patients may be more severe than supine ECMO patients. As described by Gattinoni et al., worsening patients progress from type 1 to type 2 (higher percentage of non-aerated tissue) [1], which is associated with a higher mortality rate [4]. Prone ECMO patients had much more consolidations, obviously because ECL was the main indication to be prone (*n* = 10/14). Furthermore, prone ECMO patients need a higher respiratory rate for a higher sweep gas flow suggesting that they may be exposed to a higher mechanical power, and they possibly had also a higher dead space. Second, postmortem biopsies, performed in 6 patients with ECL in the prone ECMO group, found a fibrin exudative presence both in the alveolar spaces and bronchioles followed by a fibroblastic phase [5] and raise the question of the use of corticosteroids (only one patient in the prone ECMO group). Third, as already described by Zeng et al. [6], more than half (8/11) of the patients died from septic shock and multiple organ failure, for which ECMO may be useless.

## Conclusion

Prone positioning under VV-ECMO improves oxygenation in SARS-CoV-2-induced ARDS without compromising the safety of the patients. The high mortality rate in prone ECMO patients may be explained by the greater illness severity and the lack of an immunomodulatory therapy such as corticosteroids.

## Data Availability

The datasets used and/or analyzed during the current study are available from the corresponding author on reasonable request.

## Abbreviations

ARDS: Acute respiratory distress syndrome
ICU: Intensive care unit
PP: Prone positioning
VV-ECMO: Veno-venous extracorporeal membrane oxygenation
SARS-CoV-2: Severe acute respiratory syndrome coronavirus 2
FiO_2_: Fraction of inspired oxygen
PaO_2_: Arterial partial pressure of oxygen
FDO_2_: Fraction on oxygen delivered in the sweep gas
ECL: Extensive lung consolidation
